# Use of Mass Cytometry to Profile Human T Cell Exhaustion

**DOI:** 10.3389/fimmu.2019.03039

**Published:** 2020-01-22

**Authors:** Frances Winkler, Bertram Bengsch

**Affiliations:** ^1^Department of Medicine II, Gastroenterology, Hepatology, Endocrinology, and Infectious Diseases, Faculty of Medicine, University Medical Center Freiburg, Freiburg, Germany; ^2^Faculty of Biology, University of Freiburg, Freiburg, Germany; ^3^Signalling Research Centres BIOSS and CIBSS, University of Freiburg, Freiburg, Germany

**Keywords:** T cell differentiation, systems immunology, mass cytometry (CyTOF), T cell exhaustion, chronic infections, cancer, immune checkpoint blockade, immunotherapy

## Abstract

Mass cytometry has become an important technique for the deep analysis of single cell protein expression required for precision systems immunology. The ability to profile more than 40 markers per cell is particularly relevant for the differentiation of cell types for which low parametric characterization has proven difficult, such as exhausted CD8^+^ T cells (T_EX_). T_EX_ with limited effector function accumulate in many chronic infections and cancers and are subject to inhibitory signaling mediated by several immune checkpoints (e.g., PD-1). Of note, T_EX_ represent considerable targets for immune-stimulatory therapies and are beginning to be recognized as a major correlate of successful checkpoint blockade approaches targeting the PD-1 pathway. T_EX_ exhibit substantial functional, transcriptomic and epigenomic differences compared to canonical functional T cell subsets [such as naïve (T_N_), effector (T_EFF_) and memory T cells (T_MEM_)]. However, phenotypic distinction of T_EX_ from T_EFF_ and T_MEM_ can often be challenging since many molecules expressed by T_EX_ can also be expressed by effector and memory T cell populations. Moreover, significant heterogeneity of T_EX_ has been described, such as subpopulations of exhausted T cells with progenitor-progeny relationships or populations with different degrees of exhaustion or homeostatic potential that may directly inform about disease progression. In addition, T_EX_ subsets have essential clinical implications as they differentially respond to antiviral and checkpoint therapies. The precise assessment of T_EX_ thus requires a high-parametric analysis that accounts for differences to canonical T cell populations as well as for T_EX_ subset heterogeneity. In this review, we discuss how mass cytometry can be used to reveal the role of T_EX_ subsets in humans by combining exhaustion-directed phenotyping with functional profiling. Mass cytometry analysis of human T_EX_ populations is instrumental to gain a better understanding of T_EX_ in chronic infections and cancer. It has important implications for immune monitoring in therapeutic settings aiming to boost T cell immunity, such as during cancer immunotherapy.

## Introduction

Mass cytometry has become a transformative technology for human immune cell profiling. The use of purified metal isotopes as labels for specific antibodies to stain individual cells and detection of these label isotopes on ionized cells by time-of-flight mass spectroscopy allows the analysis of the protein expression of >40 insightful markers on single cells. The lack of relevant spectral overlap of metal isotopes is a major advantage over traditional fluorescence-based flow cytometry, in which multiplexing of reagents is frequently limited by the need to compensate for overlapping emission spectra of different fluorophores. The ability to integrate the information from more than 40 detection channels for single-cell profiling has been particularly valuable for comprehensive immune monitoring (i.e., analysis of many immune cell lineages) in the setting of translational studies that involve patient cohorts with limited sample access. However, in addition to this “horizontal” profiling approach, mass cytometry also represents a key tool suitable for deep “vertical” profiling of a given immune cell population and may reveal previously unknown heterogeneity within this population, such as complexity within CD8^+^ T cells (1). In this review, we will discuss how deep immune profiling of exhausted CD8^+^ T cells by mass cytometry has led to significant insights into their heterogeneity and role in pathophysiology across chronic infections and disease. Characterization of exhausted T cells using mass cytometry is of particular relevance in many immuno-oncologic trials that aim to enhance T cell function.

## T Cell Exhaustion: Background and Main Concepts

Exhausted T cells (T_EX_) are increasingly recognized as a distinct T cell population with a key role in many chronic infections and cancer. T_EX_ were initially described in chronic viral infection, and many subsequent reports have highlighted the accumulation of T_EX_ in the context of ongoing bacterial and parasitic infection, as well as cancer and autoimmunity (2). T_EX_ are characterized by the co-expression of inhibitory receptors and reduced effector function preventing optimal control of viral infection or tumor progression. Targeting inhibitory signaling, such as by interference with inhibitory receptor PD-1 signaling or other immune checkpoints, can reinvigorate T_EX_ function and contribute to disease control or elimination. Consequently, T_EX_ have recently been identified as a major correlate of the clinical response of patients undergoing checkpoint therapy (3, 4), highlighting the need for better immune profiling of T_EX_ as a relevant biomarker for immune therapy trials.

**Figure 1:**
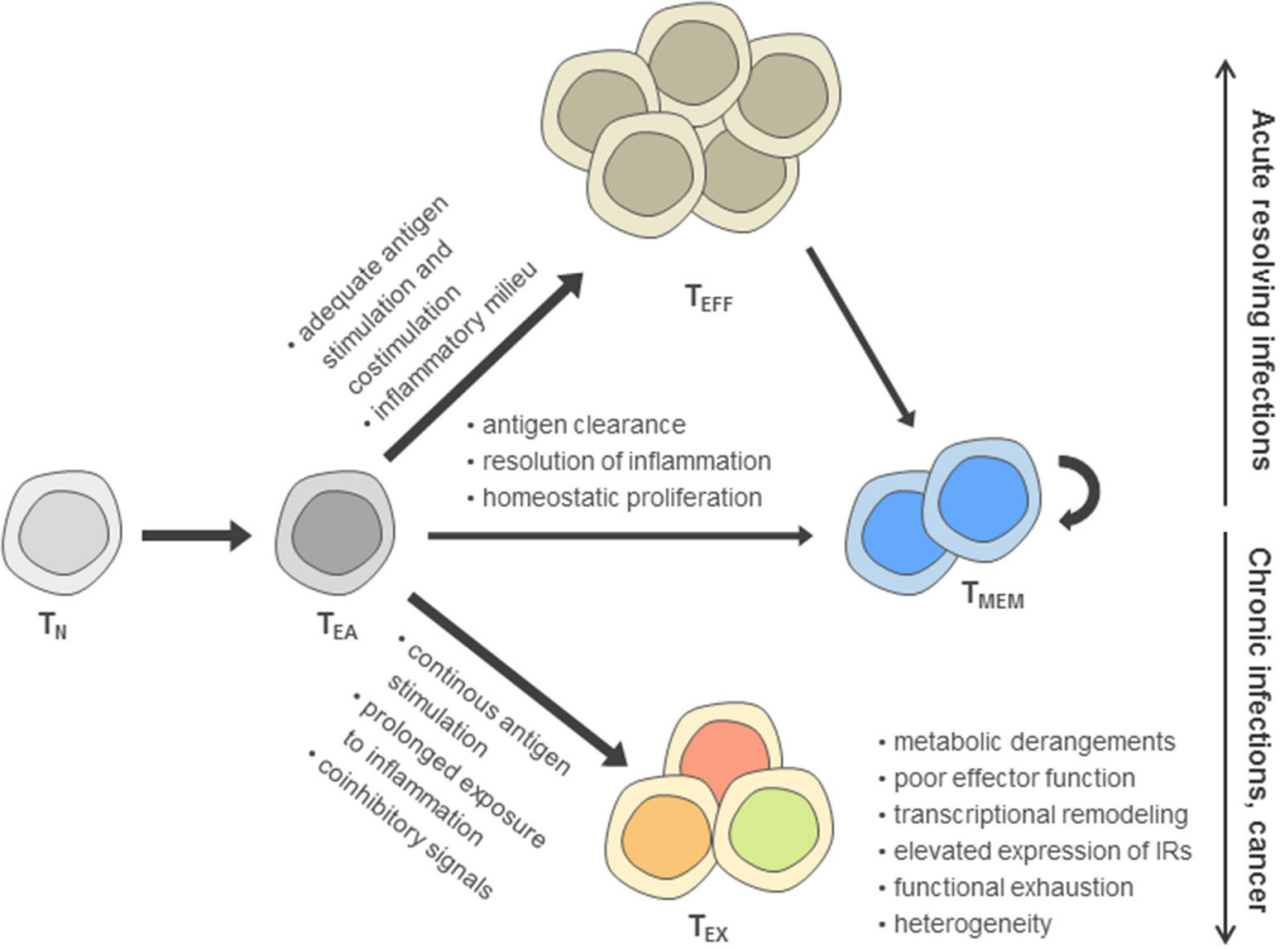
Model of post-thymic CD8^+^ T cell differentiation. According to this model, after activation of naïve T cells (T_N_) during priming, early activated effector T cells (T_EA_) receive signals driving functional differentiation to effector T cells (T_EFF_) and memory T cells (T_MEM_) depending on the recognition of antigen, costimulation, and the inflammatory milieu. In contrast, persistent antigen stimulation, reduced costimulation in the presence of coinhibitory signals and prolonged exposure to inflammatory cues are main drivers of the differentiation toward the exhausted T cell (T_EX_) fate, including an up-regulation of inhibitory receptors (IRs).

Based on the reduced effector function due to inhibitory signaling in T_EX_ compared to canonical effector T cells (T_EFF_), T_EX_ have been perceived long-term as a population of suppressed effector T cells according to a “loss-of-function” model (5–7). However, in recent years, it has become clear that the signals inducing T cell exhaustion following T cell activation can drive these cells dynamically into a distinct differentiation fate compared to T_EFF_ and memory T cells (T_MEM_) that is characterized by massive changes in their metabolism, transcriptome, and epigenome (8–16) (Figure 1).

Exhausted T cells as well as functional effector and memory T cell differentiation are most thoroughly characterized in the Lymphocytic choriomeningitis virus (LCMV) models of acute and chronic viral infection. In these models, genetic differences between acute and chronic LCMV strains are minimal and immunodominant T cell epitopes are identical, facilitating cross-comparison of T cell phenotypes at the level of endogenous responses or by analysis of transferred virus-specific T cells sharing the same T cell receptor. During acute resolving viral infection, viral clearance after induction of functional effector T cells is followed by the establishment of a pool of memory T cells (17, 18) (Figure 1). In sharp contrast, antigen-specific T cells during chronic infection progressively develop major features of exhaustion, including the up-regulation of inhibitory receptor expression and functional impairment (e.g., consecutive loss of IL-2, TNF and IFN-γ production) (6, 7, 19, 20). While in the first week of chronic infection, the exhaustion program appears to remain flexible and can be altered, as evidenced after transfer of T cells into non-chronically infected hosts, exhaustion appears to become more fixed following the second week of infection (21, 22). Notably, at later time points, the global differentiation program of T_EX_ assessed on the transcriptional and epigenetic level remains stable even after checkpoint blockade intervention and functional reinvigoration (12).

## Challenges for the Assessment of Exhausted T Cells in Humans

Many insights into T_EX_ have been obtained by the study of antigen-specific T cells in chronic infection and cancer, and multiplexed tetramer analysis together with surface and intracellular markers by mass cytometry has allowed important insights into antigen-specific T cells (1, 23). Identification of T_EX_ without prior knowledge of antigen specificity has remained challenging and is a major obstacle for immune phenotyping in human disease—in particular in cancer—where many tumor antigens especially neoantigens are unknown and tools for assessment of antigen-specific T cell populations are limited. Moreover, established models for T cell differentiation in humans based on markers such as CCR7/CD62L, CD45RA/CD45RO, or CD27/CD28 that allow assessment of naïve T cells (T_N_), T_EFF_, and T_MEM_ and additional subpopulations (e.g., central and effector memory T cells) cannot reliably distinguish T_EX_ from the effector or effector memory T cell phenotype (24). A likely explanation is that T_EX_, similar to T_EFF_, initially undergo T cell activation programs that include the downregulation of markers of naïvety (such as lymph node homing markers CCR7 and CD62L or preferential CD45RA to CD45RO alternative splicing linked to activation and memory programs), while activation markers, such as CD38 are also induced.

Many investigators have therefore turned to the profiling of inhibitory receptors with a relevant role in T_EX_ biology, such as PD-1 (alone or in combination with other inhibitory receptors such as CTLA-4, Tim-3, Lag-3, 2B4, CD160, TIGIT, and others) for the assessment of exhausted T cells in chronic infections and cancer (20, 25–35). However, it has become clear that those inhibitory receptors can also be expressed by functional effector T cells and may also be present on memory T cell populations and therefore lack specificity (36–39). Moreover, heterogeneous subpopulations of T_EX_ exist for which progenitor-progeny relationships or partial expression of memory-related programs has been described that present further challenges for phenotypic characterization (40–44). Nevertheless, while no single phenotypic marker can reliably assess T_EX_ in humans, the utility of combining several inhibitory receptors for the analysis of differences in antigen-specific exhausted T cells suggests that a combinatorial strategy integrating several exhaustion markers might overcome the limitations for immune profiling.

## Selection Strategy for Suitable Markers to Identify T_EX_ Using Mass Cytometry

Markers convenient for the identification of T_EX_ and discrimination from T_N_, T_EFF_, and T_MEM_ cells display different levels of expression on T_EX_ compared to these canonical functional T cell populations and across several disease models. To identify such appropriate exhaustion-specific markers, differences in the transcriptional and epigenomic programs between canonical T cell populations and exhausted T cells from the well-controlled LCMV model can be harnessed as specific candidate genes.

Following such an approach, sets of epigenomically regulated exhaustion-specific genes have been recently identified, including 313 genes specifically up-regulated in T_EX_ compared to T_N_, T_EFF_, and T_MEM_ that displayed higher gene expression by transcriptome analysis and concomitant changes in enhancer accessibility (45). Moreover, 182 genes down-regulated in T_EX_ were identified that were specifically suppressed on a transcriptional level and lacked accessibility of adjacent enhancers. These exhaustion-specific genes identified in the LCMV infection model were thus predicted as sufficient markers of T_EX_. The authors then validated individual genes by comparing them for their enrichment in other murine and human settings of infection and cancer, for which T cell exhaustion has been described. Specific genes for exhaustion-directed immune profiling with strong enrichment of gene expression in multiple settings of exhaustion for which suitable reagents were available for cytometry were then selected for further analysis (Table 1).

**Table 1:** Exhaustion markers for T_EX_ profiling.

**Exhaustion markers**	**Predicted expression vs. T_**N**_ T_**EFF**_ T_**MEM**_**	**Functional role**	**Minimal exhaustion panel**
2B4	UP	Co-regulatory receptor	X
Amphiregulin	UP	Cytokine	
CCL3	UP	Chemokine	
CCR7	DN	Chemokine receptor	
CD38	UP	Ectoenzyme	
CD39	UP	Ectoenzyme	X
CD7	UP	Co-regulatory receptor	
CD73	DN	Ectoenzyme	
CD127	DN	Interleukin receptor	X
CTLA-4	UP	Co-regulatory receptor	X
CXCL10	UP	Chemokine	
CXCR5	UP	Chemokine receptor	X
Eomes	UP	Transcription factor	X
Granzyme K	UP	Cytotoxic molecule	
Helios	UP	Transcription factor	
IFN-γ	ns	Cytokine	
IL-2	DN	Cytokine	
IL-10	UP	Cytokine	
IL-21	UP	Cytokine	
Lag-3	UP	Co-regulatory receptor	
PD-1	UP	Co-regulatory receptor	X
Ptger2	UP	Prostaglandin receptor	
TCF1	DN	Transcription factor	X
TIGIT	UP	Co-regulatory receptor	X
TNF	ns	Cytokine	X
TOX	UP	Transcription factor	X
XCL-1	UP	Chemokine	

*Markers were selected based on exhaustion-specific expression patterns using transcriptomic and epigenomic profiling and validated using mass cytometry. Markers associated with T cell exhaustion, their predicted expression on T_EX_ compared to other cell populations, their functional role as well as their utility for a minimal exhaustion panel is noted. Moreover, cytokines required for the assessment of T_EX_ function are included*.

This approach confirmed several markers of exhausted T cells frequently used for the profiling of T_EX_, such as inhibitory receptors PD-1, 2B4, Lag-3, TIGIT, or transcription factor Eomes. Interestingly, CD38 and CD39, which are also frequently used as activation markers due to their induction on T_EFF_ cells, displayed further exhaustion-specific up-regulation and enhancer changes compared with functional T cell populations. These observations suggest that the interpretation as activation markers indicative for T_EFF_ cells may need to be reevaluated. Furthermore, this analytic approach also identified additional exhaustion markers induced on T_EX_, such as surface proteins CD7, CXCR5, cytotoxic molecule granzyme K or transcription factors Helios and TOX, many of which are also found enriched in tumor-infiltrating lymphocytes by single-cell transcriptomics (46, 47). In agreement with the high levels of TOX on exhausted T cell populations, TOX was recently identified as a master regulator of exhaustion required for the longevity and persistence of exhausted T cells that acts via epigenetic mechanisms facilitating the expression of exhaustion-related gene programs (48, 49). In addition to these novel markers of T_EX_, it has to be noted that other molecules which are also frequently expressed by T_EX_ were not identified as exhaustion-specific candidates by this approach, including inhibitory receptors Tim-3, KLRG1, CD160, or transcription factor T-bet. This was due to lack of significant differences to canonical T cell subsets at the level of gene expression or associated enhancer changes. Similarly, additional immunoregulatory molecules such as CD72 and CD100 which have been previously described as linked to T cell exhaustion have not been identified by this pipeline, suggesting reduced specificity across T cell populations or context-dependent roles (50). It has to be noted that in this analysis of the specific expression patterns on exhausted cells, individual “exhaustion-specific” molecules can still be expressed to some degree on other T cell subsets (although with a significantly different expression level). Moreover, this strategy also predicted down-regulation of markers associated with naïve and/or memory T cells, such as CCR7, CD73, CD127 and transcription factor TCF-1 on T_EX_ (Table 1). The integration of a high number of phenotypic “exhaustion-specific” markers into mass cytometry analysis is expected to allow a better discrimination of T_EX_ populations from T_N_, T_EFF_, or T_MEM_.

## Functional Assessment of T_EX_ on a Single-Cell or Population Level

Functional impairment is a key characteristic of exhausted T cells. Indeed, the term exhaustion was initially used to describe complete loss of effector function and disappearance of the antigen-specific CD8^+^ T cell response (5, 51). However, it has since become clear that in comparison to functional effector and memory T cells, T_EX_ frequently experience a more gradual loss of effector function that can range from mild impairments in antiviral cytokine production to complete deletion. Typically, mildly exhausted cells exhibit impaired ability to produce IL-2, followed by loss of TNF production in more severe exhaustion, while the ability to produce IFN-γ is frequently maintained and lost usually only in severe exhaustion (52). Reduced expression of anti-apoptotic molecules (i.e., Bcl2) and higher levels of pro-apoptotic Bim have been reported in T_EX_ and might be linked to a pre-apoptotic phenotype in more severely exhausted cells (53–55). Moreover, reduced cytotoxicity and impaired proliferation have been announced for T_EX_, and successful reinvigoration by checkpoint blockade is frequently measured using metrics of cell cycle activity and proliferation (7). However, it has to be noted that T cell exhaustion is not simply a “loss-of-function” phenotype affecting all T cell functions. On the contrary, higher induction of some chemokines, such as CCL3 and XCL-1, and higher message of other cytokines, such as IL-10 or IL-21, by T_EX_ has been reported (8, 45, 56).

Mass cytometry has been instrumental in the comprehensive characterization of T_EX_ function. For example, differences in the cytotoxic program of T_EX_ with an increased expression of granzyme K, but reduced granzyme B and perforin can be readily assessed in combination with phenotypic profiling (57). Similarly, cell cycle activity assessed by Ki-67 combined with exhaustion marker phenotyping has been pivotal in mass cytometry analysis of responding T_EX_ during checkpoint therapies (3). Nevertheless, the unbiased per-cell assessment of complex exhaustion-related patterns of impaired cytokine production with phenotypic analysis has remained challenging. For example, analyses focusing on the ability of T cells to express effector cytokines frequently struggle to differentiate between cells that never expressed those molecules (i.e., antigen-naïve T cells) or those that lost expression (including T_EX_). To address these challenges, the characteristic impairment of polyfunctionality with regards to cytokine (e.g., IFN-γ, TNF, IL-2) but increased chemokine production (e.g., CCL3/4, XCL-1) can be used to rate individual T cells for their functional chemokine/cytokine exhaustion profile on a single-cell level using a function-passed exhaustion score. Combined with a comprehensive phenotypic exhaustion profiling possible through the use of mass cytometry, the integration of T_EX_ function as a separate metric of T cell exhaustion was able to reliably discriminate T_EX_ from T_EFF_, T_N_, and T_MEM_ (45). The combination of high-parametric functional and phenotypic exhaustion profiling may thus represent a helpful tool for the assessment of individual T_EX_ populations but also for the general degree of CD8^+^ T cell immune dysfunction in chronic disease.

## Identification of Clinically Relevant High-Dimensional T_EX_ Phenotypes Using Mass Cytometry

**Figure 2:**
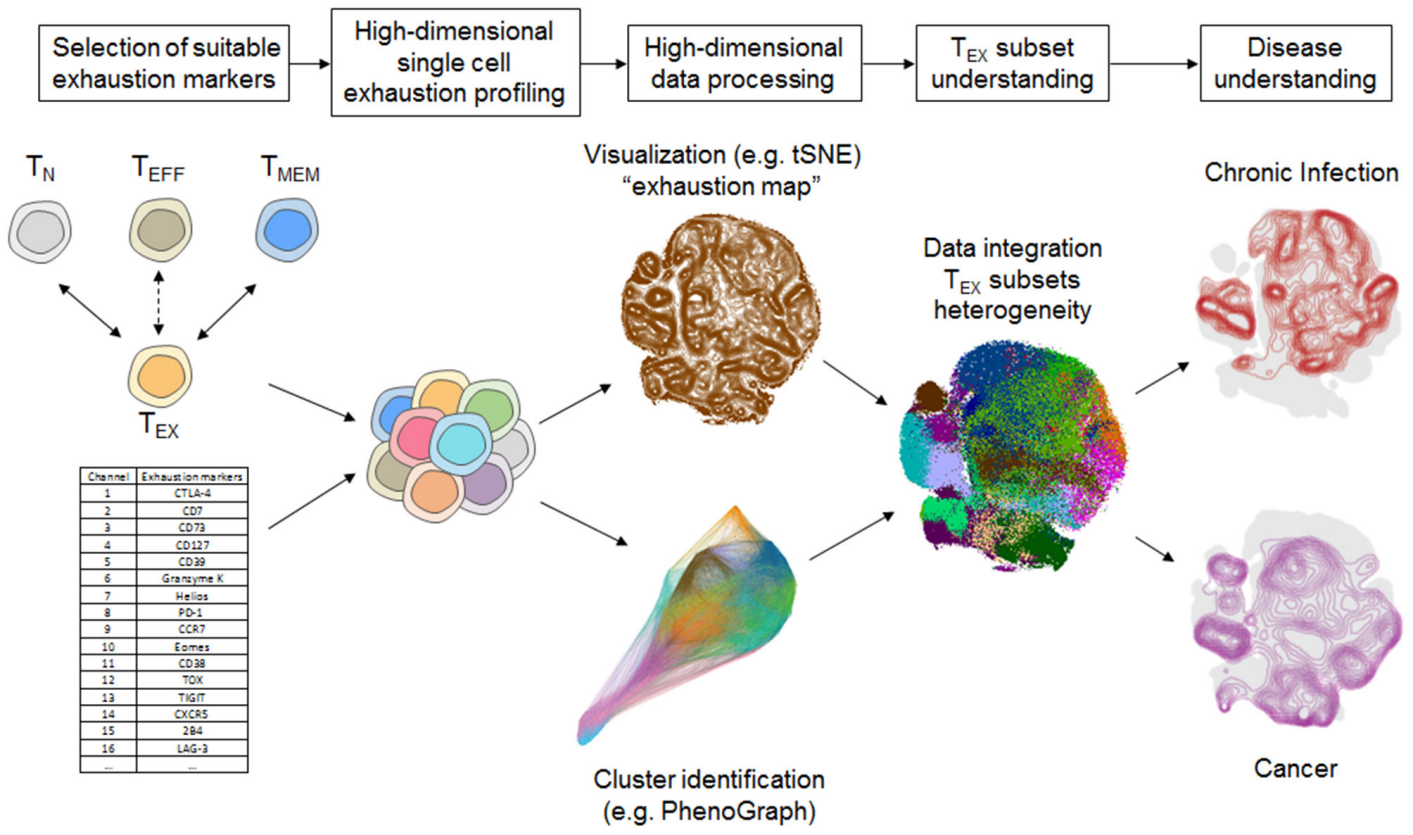
Systems immunology approach for T_EX_ characterization using mass cytometry. Selection of suitable exhaustion markers able to differentiate T_EX_ from T_EFF_ and T_MEM_ such as by transcriptomic or epigenomic profiling is a critical step for T_EX_-directed immune profiling using mass cytometry. High-dimensional analysis of T_EX_ provides further insights into the heterogeneity of T_EX_ that can be unraveled by a bioinformatics pipeline including cluster identification and dimension reduction strategies (i.e., tSNE, Phenograph) that provide detailed overview about the exhaustion landscape. Detailed T_EX_ subset profiling provides the basis for understanding the heterogeneity of T_EX_ and their involvement in different disease settings, such as chronic infection and cancer. Data-driven visualizations in this figure were computed based on a dataset published in Bengsch et al. (12).

The integration of a larger set of exhaustion markers in mass cytometry panels creates novel challenges in data evaluation. In flow cytometry-based studies with few exhaustion markers, data evaluation relies heavily on manual gating and boolean analysis of inhibitory receptor co-expression or polyfunctionality (e.g., using SPICE software analysis) (29, 58, 59). These approaches remain valuable for the assessment of T_EX_ using mass cytometry, but have disadvantages compared to bioinformatics algorithm-aided pipelines suitable for the higher data dimensionality generated by mass cytometry. Several bioinformatic strategies have been developed that allow more intuitive visualization of the high-dimensional data using dimension reduction approaches [most prominently based on visualization of “t stochastic neighborhood embedding,” tSNE (60)], and cluster identification strategies in high-dimensional data [e.g., SPADE (61), FlowSOM (62), PhenoGraph (63) and many more] or trajectory inferences that are reviewed elsewhere (64–66). For example, tSNE can be used to generate an “exhaustion map” by calculating a two-dimensional representation of the high-dimensional complexity of T_EX_ phenotypes based on exhaustion marker expression (Figure 2). This approach helps in the identification of T_EX_ heterogeneity and points toward differences of the exhaustion landscape in clinical settings (Figure 2), including the detection of specific populations of exhausted T cells enriched in the tumor microenvironment (45). Moreover, one-dimensional tSNE implementation (“OneSense”) has been used to reduce high-dimensional exhaustion phenotypes to a single parameter and compare them with other sets of marker categories (such as “function”) (67, 68). However, despite the advantages of tSNE-based analysis and high accuracy regarding local neighborhood relationships, tSNE performs different transformations on different regions of a map, resulting in possible challenges regarding interpretation of distance relationships on a tSNE map. Thus, use of tSNE for the discovery of discrete high-dimensional clusters as a crucial correlate of subsets with distinct biology is challenging. Other dimension reduction strategies with manifold approximation, such as uMAP, may address some of these limitations, but loss of information inherent to dimension-reduction strategies cannot be completely avoided (69). As a consequence, cluster identification based on the complete high-dimensional data is often preferred. For example, 25 high-dimensional clusters of CD8^+^ T cells were identified using PhenoGraph analysis based on the analysis of exhaustion markers in a large and diverse cohort of patients with chronic HIV infection, lung cancer and healthy controls (45). These clusters often projected to discrete regions of a tSNE exhaustion map that was calculated using the same exhaustion parameters but also displayed cluster affiliations that were not obvious from a tSNE map (Figure 2). Clearly, such an exhaustion marker-based clustering approach will also identify functional T cells, such as T_N_, T_EFF_, or T_MEM_ cells, but will have increased granularity for T_EX_ subset identification.

The characterization of discrete T cell clusters in high-dimensional “exhaustion data space” thus serves as a foundation that requires further detailed analysis of their functional and clinical role. In particular, a combination with functional profiling following short term stimulation is valuable to assess the extent of cellular exhaustion and can be used to determine the single-cell chemokine or cytokine pattern necessary for calculation of a “functional exhaustion score” discussed above. Such a functional profiling combined with a scaffold of phenotypic markers allowed appropriate mapping of chemokine/cytokine functionality to the high-dimensional exhaustion clusters, suggesting that 9–12 of the 25 phenotypically defined discrete CD8^+^ T cell subpopulations fit functional properties of T cell exhaustion (45).

**Figure 3:**
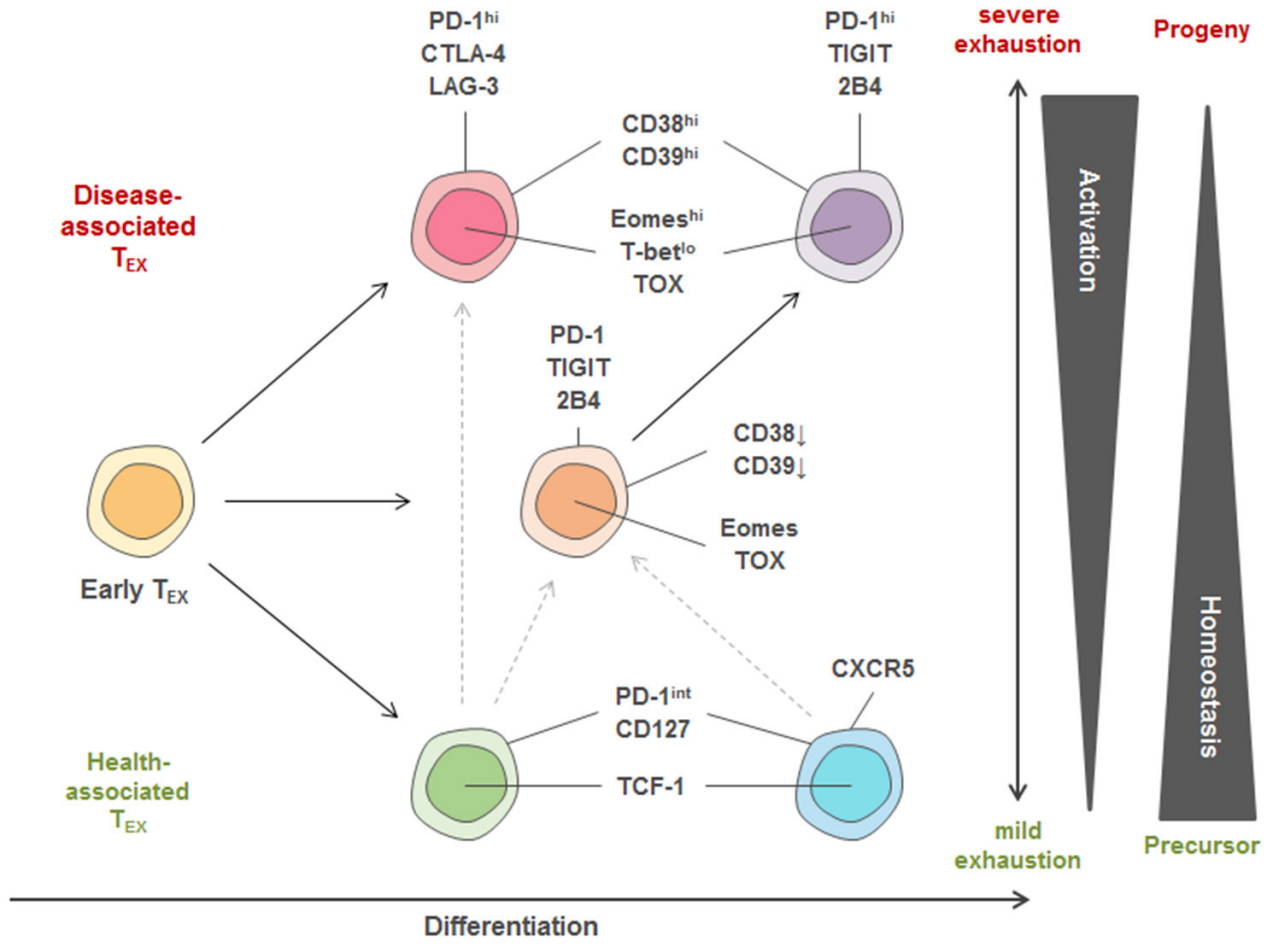
Model of T_EX_ heterogeneity and key markers linked to individual subsets. Within the pool of exhausted T cells, three major trajectories of T_EX_ subsets are proposed. Early T_EX_ can give rise to a pool of disease-associated or health-associated T_EX_ that massively differ in their activation program as well as in their transcriptional signature, while between both extremes, a balanced pool of differentiated T_EX_ can be observed. One differentiation trajectory leads to populations with high homeostatic potential that are identified in settings of disease control (“health-associated T_EX_”) and can have memory-like features, such as high TCF-1 and CD127 expression. Strong expression of activation markers also found on T_EFF_ cells (e.g., CD38, CD39) and co-expression of many inhibitory receptors (IRs) is a key feature of T_EX_ populations identified in progressive disease in chronic infection and cancer. According to this model, T_EX_ with recent history of activation and severe exhaustion after priming express a different set of IRs (e.g., PD-1, CTLA-4, Lag-3) more frequently observed in cancer compared to highly activated T cells arising in many chronic infections. These highly activated T cells in chronic infection are thought to arise from an intermediate trajectory of T_EX_ (expressing e.g., PD-1, 2B4, TIGIT) after encountering additional antigen stimulation and inhibitory signals. Furthermore, a precursor-progenitor relationship between health- and disease-associated T_EX_ important for cancer immunotherapy has been described, and is indicated by the dotted lines. The different T_EX_ trajectories also reflect differential transcriptional programming by varying T-bet, TCF-1, TOX and Eomes expression.

Several of the identified exhausted clusters enriched in severe disease contexts, such as severe HIV infection with possible AIDS, or in the tumor microenvironment of lung cancer patients. Phenotypically, these disease-associated exhausted T cells displayed co-expression of exhaustion-specific receptors such as PD-1, CD38 and a transcription factor signature characterized by high Eomes and TOX but low TCF-1 expression (Figure 3). Interestingly, disease-associated T_EX_ in chronic infection were further characterized by co-expression of inhibitory receptors TIGIT and 2B4 (as well as some KLRG1 and CD160) while in cancer, T_EX_ more frequently exhibited higher expression of CTLA-4, Lag-3, and CD39. These data suggest a conserved biology of exhausted T cells in chronic infection and cancer but also highlight specific differences in exhaustion programs with potentially large translational relevance. For example, the altered co-expression patterns of immuno-regulatory molecules on different T_EX_ populations across different disease entities or even across different stages of disease suggest that the therapeutic efficacy of combination therapies (i.e., combined targeting of PD-1 and CTLA-4) could vary according to the T_EX_ subset composition. Importantly, not all subsets of functionally exhausted T cells enrich with disease progression. For example, subsets with expression of PD-1 and co-expression of CD127, TCF-1, and CXCR5 were found enriched in HIV patients with relatively good disease control (i.e., high CD4 counts, CD4/CD8 ratio and low viral load) and these “health-associated” T_EX_ subsets were also detected in large amounts in tumor-surrounding macroscopically non-infiltrated lung compared to the tumor tissue.

These T_EX_ subpopulations identified by high-dimensional analysis using mass cytometry are in agreement with reports about severely exhausted T cells co-expressing several inhibitory receptors being linked to severe disease in chronic infection and cancer in HIV, HCV, HBV, and melanoma patients (29–32, 70–75). They are also in agreement with findings of progenitor and progeny relationships within exhausted T cell populations based on PD-1, Eomes and T-bet expression and the enrichment of T_EX_ populations with high homeostatic potential expressing TCF-1 and CD127 in scenarios of disease control (e.g., clearance of HCV infection) (40, 44, 76). Moreover, T_EX_ expressing CXCR5 have been linked to better control of HIV infection and are thought to constitute a major subset responding to checkpoint therapy (41, 42, 77). Precursor populations of exhausted tumor-infiltrating T cells with higher TCF-1 and CXCR5 expression were also identified to be linked to better clinical outcomes after checkpoint blockade therapy (78).

Thus, mass cytometry is perfectly suited for the interrogation of the clinically significant T_EX_ heterogeneity. A model of T_EX_ heterogeneity including potential lineage relationships and the suggested markers required for minimal T_EX_ immunoprofiling via mass cytometry is displayed in Figure 3 and Table 1.

## T_EX_ Profiling by Mass Cytometry: Insights for Checkpoint Therapy Monitoring

T_EX_ are emerging as a central correlate and useful biomarker of successful immune checkpoint blockade therapies. In clinical trials with patients receiving checkpoint therapies, special attention has to be directed to immune-profiling panels, as therapeutic antibodies and staining reagents may compete for the same epitope. While combination stainings with secondary antibodies against the checkpoint reagents are established, these remain to provide challenges for bioinformatic analysis. Moreover, transient permeabilization protocols frequently used for intracellular barcoding in mass cytometry trials can reduce anti-PD-1 staining. Despite these challenges, mass cytometry has been successfully applied in multiple studies profiling CD8^+^ T cells. Reinvigoration of T_EX_ compared to tumor mass was identified as a major correlate of the clinical response of patients with malign melanoma receiving checkpoint therapy with anti-PD-1 antibodies (3). Similar observations have been made using flow cytometry and in patients with non-small cell lung cancer (4). In a study comparing the differential effects of PD-1 and CTLA-4 checkpoint therapies, PD-1 blockade was found to preferentially induce a response in the CD8^+^ T_EX_ compartment, while anti-CTLA-4 therapy caused more profound changes in CD4^+^ T cells (79). These reports also highlight the utility of combining T_EX_-directed panels with markers focusing on other immune cell populations. Indeed, further evidence for the on-treatment role of T_EX_ reinvigoration during anti-PD-1 checkpoint blockade therapies came from a study that also identified a pre-treatment biomarker for checkpoint response on the level of a CD14^+^ CD16^−^ HLA-DR^+^ monocyte population (80). However, improved and more focused exhaustion-directed profiling of the heterogeneity of T_EX_ subsets will be required for a detailed understanding of T_EX_ dynamics during checkpoint therapies and personalized medicine approaches for combination therapies.

## Summary and Outlook

The inability to discriminate T_EX_ from T_EFF_ and T_MEM_ using conventional phenotyping approaches has been a longstanding problem, preventing optimal monitoring and understanding of the relevance of T_EX_ in disease. The use of high-parametric mass cytometry has been instrumental in addressing this issue and advanced the characterization of T_EX_. The combinatorial information from several exhaustion markers is required to distinguish T_EX_ from T_EFF_ and T_MEM_ and has also informed our understanding of the heterogeneity of T_EX_. One advantage of mass cytometry—the ability to integrate the higher parametric expression profile of T_EX_ with readouts for functional profiling allows the fine characterization of T_EX_ subpopulations and their involvement in human diseases. Induction of several molecular programs linked to recent T cell activation remains a shared feature of many T_EX_ populations with effector T cells (e.g., CD38, CD39, PD-1), while programs of homeostasis and access to anatomic niches can be shared by other T_EX_ populations (including those linked to disease control) with memory T cells. Heterogeneous T_EX_ subsets with different clinical roles have now been described in several translational settings of human chronic infection and cancer and are implied in differential responsiveness to immune checkpoint blockade.

Other strategies that allow an in-depth profiling of the heterogeneity of exhausted T cell populations have been described, such as scRNA-Seq, which allows the analysis of T cell transcriptomes with single cell resolution. However, currently, these approaches have several limitations over the described mass cytometry approach as they are limited by the limited sensitivity for lowly expressed genes; low cell numbers that can be practically analyzed and therefore limited ability to identify rare populations and limited scaling toward cohort analysis; lack of information on protein expression missing potential post-transcriptional regulation; and, importantly, lack of testing for T cell function. It is further unclear whether excessively larger datasets of mRNA transcripts on a single cell level will reveal more relevant heterogeneity of T_EX_ populations, as the profiling approach outlined above already was designed to maximize utilization of markers informative for differences between T_EX_ and functional T cell subsets. Of note, the combinatorial complexity of high dimensional data generated during immunome analysis by mass cytometry is different from other omics approaches. For example, simultaneous analysis of mass cytometric immune profiling together with transcriptome, microbiome, proteome and metabolome analysis during pregnancy indicated that the mass cytometry dataset was more informative based on modularity analysis (as measured by the number of principal components needed to account for 90% variance of each dataset) compared to the other omics datasets assessing significantly larger numbers of parameters (81).

To date the most detailed profiling of T_EX_ has been performed in murine models of chronic infection or cancer. With detailed resolution of T_EX_ heterogeneity in humans now accessible through the use of specialized mass cytometry analysis, more detailed identification of T_EX_ features associated with specific types of diseases and anatomical locations that will guide understanding of changes in T_EX_ cell populations in chronic infections, cancer and even autoimmunity is expected. The expression of many exhaustion-related proteins involved in many immuno-regulatory pathways amenable to therapeutic intervention also suggests that T_EX_ profiling might be required for adequate selection of combination therapies and could become indispensable for the rational design of personalized therapeutic treatments.

In sum, deep immune profiling of T_EX_ using mass cytometry is expected to provide further insights into the biology underlying this special T cell differentiation stage and its role in pathogenesis and response to immune therapies in cancer, viral infection and autoimmunity.

## Author Contributions

FW and BB conceived and wrote the manuscript, revising it critically for important intellectual content.

### Conflict of Interest

The authors declare that the research was conducted in the absence of any commercial or financial relationships that could be construed as a potential conflict of interest.

## References

[1] Ahn E Araki K Hashimoto M Li W Riley JL Cheung J. Role of PD-1 during effector CD8 T cell differentiation. Proc Natl Acad Sci USA. (2018) 115:4749–54. 10.1073/pnas.1718217115 29654146 PMC5939075

[2] Alfei F Kanev K Hofmann M Wu M Ghoneim HE Roelli P. TOX reinforces the phenotype and longevity of exhausted T cells in chronic viral infection. Nature. (2019) 571:265–9. 10.1038/s41586-019-1326-9 31207605

[3] Amir El AD Davis KL Tadmor MD Simonds EF Levine JH Bendall SC viSNE enables visualization of high dimensional single-cell data and reveals phenotypic heterogeneity of leukemia. Nat Biotechnol. (2013) 31:545–52. 10.1038/nbt.2594 23685480 PMC4076922

[4] Angelosanto JM Blackburn SD Crawford A Wherry EJ. Progressive loss of memory T cell potential and commitment to exhaustion during chronic viral infection. J Virol. (2012) 86:8161–70. 10.1128/JVI.00889-12 22623779 PMC3421680

[5] Appay V Van Lier RA Sallusto F Roederer M. Phenotype and function of human T lymphocyte subsets: consensus and issues. Cytometry A. (2008) 73:975–83. 10.1002/cyto.a.20643 18785267

[6] Baitsch L Baumgaertner P Devevre E Raghav SK Legat A Barba L. Exhaustion of tumor-specific CD8(+) T cells in metastases from melanoma patients. J Clin Invest. (2011) 121:2350–60. 10.1172/JCI46102 21555851 PMC3104769

[7] Barber DL Wherry EJ Masopust D Zhu B Allison JP Sharpe AH. Restoring function in exhausted CD8 T cells during chronic viral infection. Nature. (2006) 439:682–7. 10.1038/nature04444 16382236

[8] Becht E Mcinnes L Healy J Dutertre CA Kwok IWH Ng LG Dimensionality reduction for visualizing single-cell data using UMAP. Nat Biotechnol. (2018) 37:38–44. 10.1038/nbt.4314 30531897

[9] Bengsch B Johnson AL Kurachi M Odorizzi PM Pauken KE Attanasio J. Bioenergetic insufficiencies due to metabolic alterations regulated by the inhibitory receptor PD-1 are an early driver of CD8(+) T cell exhaustion. Immunity. (2016) 45:358–73. 10.1016/j.immuni.2016.07.008 27496729 PMC4988919

[10] Bengsch B Martin B Thimme R. Restoration of HBV-specific CD8+ T cell function by PD-1 blockade in inactive carrier patients is linked to T cell differentiation. J Hepatol. (2014) 61:1212–9. 10.1016/j.jhep.2014.07.005 25016223

[11] Bengsch B Ohtani T Herati RS Bovenschen N Chang KM Wherry EJ. Deep immune profiling by mass cytometry links human T and NK cell differentiation and cytotoxic molecule expression patterns. J Immunol Methods. (2018) 453:3–10. 10.1016/j.jim.2017.03.009 28322863 PMC5605401

[12] Bengsch B Ohtani T Khan O Setty M Manne S O'brien S. (2018). Epigenomic-guided mass cytometry profiling reveals disease-specific features of exhausted CD8 T cells. Immunity. 48, 1029–45.e1025. 10.1016/j.immuni.2018.04.026 29768164 PMC6010198

[13] Bengsch B Seigel B Ruhl M Timm J Kuntz M Blum HE. Coexpression of PD-1, 2B4, CD160 and KLRG1 on exhausted HCV-specific CD8+ T cells is linked to antigen recognition and T cell differentiation. PLoS Pathog. (2010) 6:e1000947. 10.1371/journal.ppat.1000947 20548953 PMC2883597

[14] Betts MR Nason MC West SM De Rosa SC Migueles SA Abraham J. HIV nonprogressors preferentially maintain highly functional HIV-specific CD8+ T cells. Blood. (2006) 107:4781–9. 10.1182/blood-2005-12-4818 16467198 PMC1895811

[15] Blackburn SD Shin H Haining WN Zou T Workman CJ Polley A. Coregulation of CD8+ T cell exhaustion by multiple inhibitory receptors during chronic viral infection. Nat Immunol. (2009) 10:29–37. 10.1038/ni.1679 19043418 PMC2605166

[16] Boni C Fisicaro P Valdatta C Amadei B Di Vincenzo P Giuberti T. Characterization of hepatitis B virus (HBV)-specific T-cell dysfunction in chronic HBV infection. J Virol. (2007) 81:4215–25. 10.1128/JVI.02844-06 17287266 PMC1866111

[17] Brooks DG Ha SJ Elsaesser H Sharpe AH Freeman GJ Oldstone MB. IL-10 and PD-L1 operate through distinct pathways to suppress T-cell activity during persistent viral infection. Proc Natl Acad Sci USA. (2008) 105:20428–33. 10.1073/pnas.0811139106 19075244 PMC2629263

[18] Buggert M Tauriainen J Yamamoto T Frederiksen J Ivarsson MA Michaelsson J. T-bet and Eomes are differentially linked to the exhausted phenotype of CD8+ T cells in HIV infection. PLoS Pathog. (2014) 10:e1004251. 10.1371/journal.ppat.1004251 25032686 PMC4102564

[19] Cheng Y Wong MT Van Der Maaten L Newell EW. Categorical analysis of human T cell heterogeneity with one-dimensional soli-expression by nonlinear stochastic embedding. J Immunol. (2016) 196:924–32. 10.4049/jimmunol.1501928 26667171 PMC4705595

[20] Cheng Y Zhu YO Becht E Aw P Chen J Poidinger M. Multifactorial heterogeneity of virus-specific T cells and association with the progression of human chronic hepatitis B infection. Sci Immunol. (2019) 4:eaau6905. 10.1126/sciimmunol.aau6905 30737354

[21] Chevrier S Levine JH Zanotelli VRT Silina K Schulz D Bacac M. An immune atlas of clear cell renal cell carcinoma. Cell. (2017) 169:736–49.e718. 10.1016/j.cell.2017.04.016 28475899 PMC5422211

[22] Correa-Rocha R Lopez-Abente J Gutierrez C Perez-Fernandez VA Prieto-Sanchez A Moreno-Guillen S. CD72/CD100 and PD-1/PD-L1 markers are increased on T and B cells in HIV-1+ viremic individuals, and CD72/CD100 axis is correlated with T-cell exhaustion. PLoS ONE. (2018) 13:e0203419. 10.1371/journal.pone.0203419 30161254 PMC6117071

[23] Day CL Kaufmann DE Kiepiela P Brown JA Moodley ES Reddy S. PD-1 expression on HIV-specific T cells is associated with T-cell exhaustion and disease progression. Nature. (2006) 443:350–4. 10.1038/nature05115 16921384

[24] Doering TA Crawford A Angelosanto JM Paley MA Ziegler CG Wherry EJ. Network analysis reveals centrally connected genes and pathways involved in CD8+ T cell exhaustion versus memory. Immunity. (2012) 37:1130–44. 10.1016/j.immuni.2012.08.021 23159438 PMC3749234

[25] Duraiswamy J Ibegbu CC Masopust D Miller JD Araki K Doho GH. Phenotype, function, and gene expression profiles of programmed death-1(hi) CD8 T cells in healthy human adults. J Immunol. (2011) 186:4200–12. 10.4049/jimmunol.1001783 21383243 PMC3723805

[26] Fourcade J Sun Z Benallaoua M Guillaume P Luescher IF Sander C. Upregulation of Tim-3 and PD-1 expression is associated with tumor antigen-specific CD8+ T cell dysfunction in melanoma patients. J Exp Med. (2010) 207:2175–86. 10.1084/jem.20100637 20819923 PMC2947081

[27] Fuller MJ Zajac AJ. Ablation of CD8 and CD4 T cell responses by high viral loads. J Immunol. (2003) 170:477–86. 10.4049/jimmunol.170.1.477 12496434

[28] Gehring AJ Ho ZZ Tan AT Aung MO Lee KH Tan KC. Profile of tumor antigen-specific CD8 T cells in patients with hepatitis B virus-related hepatocellular carcinoma. Gastroenterology. (2009) 137:682–90. 10.1053/j.gastro.2009.04.045 19394336

[29] Ghaemi MS Digiulio DB Contrepois K Callahan B Ngo TTM Lee-Mcmullen B. Multiomics modeling of the immunome, transcriptome, microbiome, proteome and metabolome adaptations during human pregnancy. Bioinformatics. (2019) 35:95–103. 10.1093/bioinformatics/bty537 30561547 PMC6298056

[30] Ghoneim HE Fan Y Moustaki A Abdelsamed HA Dash P Dogra P. *De novo* epigenetic programs inhibit PD-1 blockade-mediated T cell rejuvenation. Cell. (2017) 170:142–57.e19. 10.1016/j.cell.2017.06.007 28648661 PMC5568784

[31] Grayson JM Weant AE Holbrook BC Hildeman D. Role of Bim in regulating CD8+ T-cell responses during chronic viral infection. J Virol. (2006) 80:8627–38. 10.1128/JVI.00855-06 16912311 PMC1563887

[32] He R Hou S Liu C Zhang A Bai Q Han M. Follicular CXCR5-expressing CD8+ T cells curtail chronic viral infection. Nature. (2016) 537:412–28. 10.1038/nature19317 27501245

[33] Hoffmann M Pantazis N Martin GE Hickling S Hurst J Meyerowitz J. Exhaustion of activated CD8 T cells predicts disease progression in primary HIV-1 infection. PLoS Pathog. (2016) 12:e1005661. 10.1371/journal.ppat.1005661 27415828 PMC4945085

[34] Huang AC Postow MA Orlowski RJ Mick R Bengsch B Manne S. T-cell invigoration to tumour burden ratio associated with anti-PD-1 response. Nature. (2017) 545:60–5. 10.1038/nature22079 28397821 PMC5554367

[35] Im SJ Hashimoto M Gerner MY Lee J Kissick HT Burger MC. Defining CD8+ T cells that provide the proliferative burst after PD-1 therapy. Nature. (2016) 537:417–21. 10.1038/nature19330 27501248 PMC5297183

[36] Kaech SM Tan JT Wherry EJ Konieczny BT Surh CD Ahmed R. Selective expression of the interleukin 7 receptor identifies effector CD8 T cells that give rise to long-lived memory cells. Nat Immunol. (2003) 4:1191–8. 10.1038/ni1009 14625547

[37] Kamphorst AO Pillai RN Yang S Nasti TH Akondy RS Wieland A. Proliferation of PD-1+ CD8 T cells in peripheral blood after PD-1-targeted therapy in lung cancer patients. Proc Natl Acad Sci USA. (2017) 114:4993–8. 10.1073/pnas.1705327114 28446615 PMC5441721

[38] Khan O Giles JR Mcdonald S Manne S Ngiow SF Patel KP. TOX transcriptionally and epigenetically programs CD8(+) T cell exhaustion. Nature. (2019) 571:211–8. 10.1038/s41586-019-1325-x 31207603 PMC6713202

[39] Krieg C Nowicka M Guglietta S Schindler S Hartmann FJ Weber LM High-dimensional single-cell analysis predicts response to anti-PD-1 immunotherapy. Nat Med. (2018) 24:144–53. 10.1038/nm.4466 29309059

[40] Legat A Speiser DE Pircher H Zehn D Fuertes Marraco SA. Inhibitory receptor expression depends more dominantly on differentiation and activation than “exhaustion” of human CD8 T cells. Front Immunol. (2013) 4:455. 10.3389/fimmu.2013.00455 24391639 PMC3867683

[41] Levine JH Simonds EF Bendall SC Davis KL Amir El AD Tadmor MD. Data-driven phenotypic dissection of AML reveals progenitor-like cells that correlate with prognosis. Cell. (2015) 162:184–97. 10.1016/j.cell.2015.05.047 26095251 PMC4508757

[42] Lopes AR Kellam P Das A Dunn C Kwan A Turner J. Bim-mediated deletion of antigen-specific CD8 T cells in patients unable to control HBV infection. J Clin Invest. (2008) 118:1835–45. 10.1172/JCI33402 18398508 PMC2289792

[43] Mclane LM Abdel-Hakeem MS Wherry EJ. CD8 T Cell Exhaustion during chronic viral infection and cancer. Annu Rev Immunol. (2019) 37:457–95. 10.1146/annurev-immunol-041015-055318 30676822

[44] Miller BC Sen DR Al Abosy R Bi K Virkud YV Lafleur MW Subsets of exhausted CD8(+) T cells differentially mediate tumor control and respond to checkpoint blockade. Nat Immunol. (2019) 20:326–36. 10.1038/s41590-019-0312-6 30778252 PMC6673650

[45] Moskophidis D Lechner F Pircher H Zinkernagel RM. Virus persistence in acutely infected immunocompetent mice by exhaustion of antiviral cytotoxic effector T cells. Nature. (1993) 362:758–61. 10.1038/362758a0 8469287

[46] Nakamoto N Cho H Shaked A Olthoff K Valiga ME Kaminski M. Synergistic reversal of intrahepatic HCV-specific CD8 T cell exhaustion by combined PD-1/CTLA-4 blockade. PLoS Pathog. (2009) 5:e1000313. 10.1371/journal.ppat.1000313 19247441 PMC2642724

[47] Nakamoto N Kaplan DE Coleclough J Li Y Valiga ME Kaminski M. Functional restoration of HCV-specific CD8 T cells by PD-1 blockade is defined by PD-1 expression and compartmentalization. Gastroenterology. (2008) 134:1927–1937, 1937.e1–2. 10.1053/j.gastro.2008.02.033 18549878 PMC2665722

[48] Newell EW Sigal N Bendall SC Nolan GP Davis MM. Cytometry by time-of-flight shows combinatorial cytokine expression and virus-specific cell niches within a continuum of CD8+ T cell phenotypes. Immunity. (2012) 36:142–52. 10.1016/j.immuni.2012.01.002 22265676 PMC3752833

[49] Olsen LR Leipold MD Pedersen CB Maecker HT. The anatomy of single cell mass cytometry data. Cytometry A. (2019) 95:156–72. 10.1002/cyto.a.23621 30277658

[50] Paley MA Kroy DC Odorizzi PM Johnnidis JB Dolfi DV Barnett BE. Progenitor and terminal subsets of CD8+ T cells cooperate to contain chronic viral infection. Science. (2012) 338:1220–5. 10.1126/science.1229620 23197535 PMC3653769

[51] Pauken KE Sammons MA Odorizzi PM Manne S Godec J Khan O. Epigenetic stability of exhausted T cells limits durability of reinvigoration by PD-1 blockade. Science. (2016) 354:1160–1165. 10.1126/science.aaf2807 27789795 PMC5484795

[52] Penna A Pilli M Zerbini A Orlandini A Mezzadri S Sacchelli L. Dysfunction and functional restoration of HCV-specific CD8 responses in chronic hepatitis C virus infection. Hepatology. (2007) 45:588–601. 10.1002/hep.21541 17326153

[53] Petrovas C Chaon B Ambrozak DR Price DA Melenhorst JJ Hill BJ. Differential association of programmed death-1 and CD57 with *ex vivo* survival of CD8+ T cells in HIV infection. J Immunol. (2009) 183:1120–32. 10.4049/jimmunol.0900182 19564339 PMC2923541

[54] Petrovas C Ferrando-Martinez S Gerner MY Casazza JP Pegu A Deleage C. Follicular CD8 T cells accumulate in HIV infection and can kill infected cells *in vitro* via bispecific antibodies. Sci Transl Med. (2017) 9:eaag2285. 10.1126/scitranslmed.aag2285 28100833 PMC5497679

[55] Philip M Fairchild L Sun L Horste EL Camara S Shakiba M. Chromatin states define tumour-specific T cell dysfunction and reprogramming. Nature. (2017) 545:452–6. 10.1038/nature22367 28514453 PMC5693219

[56] Qiu P Simonds EF Bendall SC Gibbs KD Jr Bruggner RV Linderman MD. Extracting a cellular hierarchy from high-dimensional cytometry data with SPADE. Nat Biotechnol. (2011) 29:886–91. 10.1038/nbt.1991 21964415 PMC3196363

[57] Radziewicz H Ibegbu CC Fernandez ML Workowski KA Obideen K Wehbi M. Liver-infiltrating lymphocytes in chronic human hepatitis C virus infection display an exhausted phenotype with high levels of PD-1 and low levels of CD127 expression. J Virol. (2007) 81:2545–53. 10.1128/JVI.02021-06 17182670 PMC1865979

[58] Roederer M Nozzi JL Nason MC. SPICE: exploration and analysis of post-cytometric complex multivariate datasets. Cytometry A. (2011) 79:167–74. 10.1002/cyto.a.21015 21265010 PMC3072288

[59] Saelens W Cannoodt R Todorov H Saeys Y. A comparison of single-cell trajectory inference methods. Nat Biotechnol. (2019) 37:547–54. 10.1038/s41587-019-0071-9 30936559

[60] Sauce D Almeida JR Larsen M Haro L Autran B Freeman GJ. PD-1 expression on human CD8 T cells depends on both state of differentiation and activation status. AIDS. (2007) 21:2005–13. 10.1097/QAD.0b013e3282eee548 17885290

[61] Schietinger A Philip M Krisnawan VE Chiu EY Delrow JJ Basom RS. Tumor-specific T cell dysfunction is a dynamic antigen-driven differentiation program initiated early during tumorigenesis. Immunity. (2016) 45:389–401. 10.1016/j.immuni.2016.07.011 27521269 PMC5119632

[62] Sen DR Kaminski J Barnitz RA Kurachi M Gerdemann U Yates KB. The epigenetic landscape of T cell exhaustion. Science. (2016) 354:1165–9. 10.1126/science.aae0491 27789799 PMC5497589

[63] Simoni Y Fehlings M Newell EW. Multiplex MHC class I tetramer combined with intranuclear staining by mass cytometry. Methods Mol Biol. (2019) 1989:147–58. 10.1007/978-1-4939-9454-0_11 31077105

[64] Staron MM Gray SM Marshall HD Parish IA Chen JH Perry CJ. The transcription factor FoxO1 sustains expression of the inhibitory receptor PD-1 and survival of antiviral CD8(+) T cells during chronic infection. Immunity. (2014) 41:802–14. 10.1016/j.immuni.2014.10.013 25464856 PMC4270830

[65] Tirosh I Izar B Prakadan SM Wadsworth MH II Treacy D Trombetta JJ. Dissecting the multicellular ecosystem of metastatic melanoma by single-cell RNA-seq. Science. (2016) 352, 189–196. 10.1126/science.aad0501 27124452 PMC4944528

[66] Trautmann L Janbazian L Chomont N Said EA Gimmig S Bessette B. Upregulation of PD-1 expression on HIV-specific CD8+ T cells leads to reversible immune dysfunction. Nat Med. (2006) 12:1198–202. 10.1038/nm1482 16917489

[67] Urbani S Amadei B Tola D Massari M Schivazappa S Missale G. PD-1 expression in acute hepatitis C virus (HCV) infection is associated with HCV-specific CD8 exhaustion. J Virol. (2006) 80:11398–403. 10.1128/JVI.01177-06 16956940 PMC1642188

[68] Utzschneider DT Charmoy M Chennupati V Pousse L Ferreira DP Calderon-Copete S. T Cell factor 1-expressing memory-like CD8(+) T cells sustain the immune response to chronic viral infections. Immunity. (2016) 45:415–27. 10.1016/j.immuni.2016.07.021 27533016

[69] Utzschneider DT Legat A Fuertes Marraco SA Carrie L Luescher I Speiser DE. T cells maintain an exhausted phenotype after antigen withdrawal and population reexpansion. Nat Immunol. (2013) 14:603–10. 10.1038/ni.2606 23644506

[70] Van Gassen S Callebaut B Van Helden MJ Lambrecht BN Demeester P Dhaene T. FlowSOM: using self-organizing maps for visualization and interpretation of cytometry data. Cytometry A. (2015) 87:636–45. 10.1002/cyto.a.22625 25573116

[71] Weber LM Robinson MD. Comparison of clustering methods for high-dimensional single-cell flow and mass cytometry data. Cytometry A. (2016) 89:1084–96. 10.1002/cyto.a.23030 27992111

[72] Wei SC Levine JH Cogdill AP Zhao Y Anang NAS. Distinct cellular mechanisms underlie anti-CTLA-4 and anti-PD-1 checkpoint blockade. Cell. (2017) 170:1120–33.e1117. 10.1016/j.cell.2017.07.024 28803728 PMC5591072

[73] Wherry EJ Ahmed R. Memory CD8 T-cell differentiation during viral infection. J Virol. (2004) 78:5535–45. 10.1128/JVI.78.11.5535-5545.2004 15140950 PMC415833

[74] Wherry EJ Blattman JN Murali-Krishna K Van Der Most R Ahmed R. Viral persistence alters CD8 T-cell immunodominance and tissue distribution and results in distinct stages of functional impairment. J Virol. (2003) 77:4911–27. 10.1128/JVI.77.8.4911-4927.2003 12663797 PMC152117

[75] Wherry EJ Ha SJ Kaech SM Haining WN Sarkar S Kalia V. Molecular signature of CD8+ T cell exhaustion during chronic viral infection. Immunity. (2007) 27:670–84. 10.1016/j.immuni.2007.09.006 17950003

[76] Wieland D Kemming J Schuch A Emmerich F Knolle P Neumann-Haefelin C. TCF1(+) hepatitis C virus-specific CD8(+) T cells are maintained after cessation of chronic antigen stimulation. Nat Commun. (2017) 8:15050. 10.1038/ncomms15050 28466857 PMC5418623

[77] Yamamoto T Price DA Casazza JP Ferrari G Nason M Chattopadhyay PK. Surface expression patterns of negative regulatory molecules identify determinants of virus-specific CD8+ T-cell exhaustion in HIV infection. Blood. (2011) 117:4805–15. 10.1182/blood-2010-11-317297 21398582 PMC3100691

[78] Youngblood B Hale JS Kissick HT Ahn E Xu X Wieland A. Effector CD8 T cells dedifferentiate into long-lived memory cells. Nature. (2017) 552:404–9. 10.1038/nature25144 29236683 PMC5965677

[79] Zajac AJ Blattman JN Murali-Krishna K Sourdive DJ Suresh M Altman JD. Viral immune evasion due to persistence of activated T cells without effector function. J Exp Med. (1998) 188:2205–13. 10.1084/jem.188.12.2205 9858507 PMC2212420

[80] Zhang Z Zhang JY Wherry EJ Jin B Xu B Zou ZS. Dynamic programmed death 1 expression by virus-specific CD8 T cells correlates with the outcome of acute hepatitis B. Gastroenterology. (2008) 134:1938–49, 1949.e1–3. 10.1053/j.gastro.2008.03.037 18455515

[81] Zheng C Zheng L Yoo JK Guo H Zhang Y Guo X. Landscape of infiltrating T cells in liver cancer revealed by single-cell sequencing. Cell. (2017) 169:1342–56.e1316. 10.1016/j.cell.2017.05.035 28622514

